# Characterization and management of interaction risks between livestock and wild ungulates on outdoor pig farms in Spain

**DOI:** 10.1186/s40813-021-00246-7

**Published:** 2022-01-05

**Authors:** Saúl Jiménez-Ruiz, Eduardo Laguna, Joaquín Vicente, Ignacio García-Bocanegra, Jordi Martínez-Guijosa, David Cano-Terriza, María A. Risalde, Pelayo Acevedo

**Affiliations:** 1 Grupo Sanidad y Biotecnología (SaBio), Instituto de Investigación en Recursos Cinegéticos IREC (UCLM-CSIC-JCCM), Universidad de Castilla-La Mancha (UCLM), 13071 Ciudad Real, Spain; 2 Grupo de Investigación en Sanidad Animal y Zoonosis (GISAZ), Departamento de Sanidad Animal, Facultad de Veterinaria, Universidad de Córdoba (UCO), 14014 Córdoba, Spain; 3 Departamento de Anatomía y Anatomía Patológica Comparadas y Toxicología, Facultad de Veterinaria, Universidad de Córdoba (UCO), 14014 Córdoba, Spain; 4 Unidad de Enfermedades Infecciosas, Grupo de Virología Clínica y Zoonosis, Instituto Maimónides de Investigación Biomédica de Córdoba (IMIBIC), Hospital Reina Sofía, Universidad de Córdoba (UCO), Córdoba, Spain

**Keywords:** Outdoor farming, Wildlife-livestock interface, Risk of species interaction, Biosecurity, Standardised approach, Iberian pig, Wild boar, Red deer

## Abstract

**Background:**

To control the transmission of relevant shared diseases, such as animal tuberculosis (TB) and African swine fever (ASF), it is essential to reduce the risk of interaction between livestock and wild ungulates. In Eastern and Central Europe, the current spread of ASF virus affecting wild boar and domestic pigs (especially those raised outdoors and/or in backyards) has devastated the pig sector in affected regions and is seriously threatening other exporting countries. Here, we evaluated the risk of wildlife-livestock interactions on 45 outdoor pig farms in Spain, the second largest pork producer in the EU and then proposed biosecurity-related actions. An integrated, systematic wildlife risk mitigation protocol based on interviews, questionnaires and field audits was developed and applied on each farm.

**Results:**

Most of the interaction risk points were associated with water sources (84.2%; 701/832), mainly springs and ponds, which accounted for almost all the specific points with high or very high risk scores. The risk of interaction at feeding points (6.9%; 57/832) and those associated with facilities for livestock and/or game management (8.9%; 74/832) were rated as low and very low risk, respectively. Wild boar were present and hunted on 69% of the farms. Supplementary feeding for wild ungulate species (mainly wild boar) was provided on almost half (48.9%; 22/45) the surveyed farms. Risk mitigation actions were categorised to target water access, waterers, food, other livestock species, grazing, wildlife, and offal disposal. Of the total number of actions (n = 2016), 82.7% were identified as priority actions while 17.3% represented alternative options which were identified less cost-effective. On average, 37.1 (median: 32; range 14–113) action proposals per study farm were made and 2.0 (median: 1; range 0–4) per risk point. The mean estimated cost of implementing the proposed priority actions was 14,780 €/farm (25.7 €/hectare and 799.4 €/risk point).

**Conclusions:**

This study expands the knowledge of interaction risks between domestic pigs and wild ungulates in outdoor pig farming systems and highlights the importance of considering local risks and management practices when designing and prioritising adapted wildlife risk mitigation and biosecurity actions. This practical and feasible protocol developed for Mediterranean ecosystems is easily transferable to professionals and can be adapted to extensive (outdoor) production or epidemiological systems in other European regions.

**Supplementary Information:**

The online version contains supplementary material available at 10.1186/s40813-021-00246-7.

## Background

Wild ungulate populations have increased considerably in number and distribution in Europe over the last few decades, affecting the functioning of natural ecosystems [1, 2]. These populations have also expanded spatially, often overlapping with areas of livestock production [3]. In such scenarios, the opportunities for interspecies interactions at the wildlife-livestock interface increase, as does the risk of maintenance and spread of shared pathogens [4]. This situation is of particular concern in extensive production systems where domestic and wild species share resources and interspecies transmission of multi-host pathogens can be favoured [5]. The most important infections shared at the wildlife-livestock interface were categorised globally in the early 2000s [6]. In Europe, animal tuberculosis (TB) and African swine fever (ASF) are currently among the shared infections involving wild ungulates most difficult to control [7, 8].

In Mediterranean environments, recent research has focused on describing patterns of land use of wild ungulates in outdoor farming areas to gain a better understanding of the risks for pathogen transmission at the wildlife-livestock interface [9–14]. These studies have highlighted that (1) indirect interactions between wild and domestic ungulates are more significant than direct ones, (2) the availability of natural resources (mainly water, but also food) is an important risk factor for the occurrence of interactions, (3) the times of greatest risk are late summer-early autumn and twilight, and (4) the (over)abundance of wild ungulates and livestock overload are important determinants of the frequency of interspecific interactions. The occurrence of several shared infections at the wildlife-livestock interface has also been surveyed in this scenario, supporting previous ecological and epidemiological findings [15–17].

Different management actions, such as movement restrictions [18], controlling wild ungulate populations through culling [19, 20] and/or improving farming practices [21] can mitigate potential interactions at the wildlife-livestock interface. However, standardised farm-specific wildlife risk mitigation protocols are still in their infancy [8, but see 22]. Extensive animal production systems (e.g., outdoor pig farming in Europe; see below) are not always fully standardised and implementation of biosecurity measures is based more on farmers’ perceptions [23] and socio-economic factors [24] than on scientific evidence [25].

Pig production is an important economic driver in many countries, since it accounts for around 30% of meat consumption worldwide [26]. The European Union (EU) is the world’s second biggest producer of pork and the leading exporter of pork and pork products, respectively [27]. Outdoor pig farming accounts for over 16% of the total number of European pig farms and around 0.7% of the total number of pigs [8]. However, even though this type of pig production has increased in recent decades due to animal welfare issues and environmentally friendly products [28], there is neither legislation nor guidelines for standardisation of this production system at the European level [8]. In Spain, the second biggest pork producer in the EU [27], outdoor production accounts for 17% of the total number of farms (5.1% of the total number of pigs), mostly belonging to the Iberian pig breed and its crossbreeds [8]. Approximately 80% of Iberian pig farms are in southwestern Spain, where the main representative landscape is the *dehesa* (5.8 million ha) [29] and pigs are raised under extensive management conditions where the production cycle mostly ends with fattening by feeding on acorns. This ecological system is usually interspersed with Mediterranean forest and scrublands where wild ungulate populations, mainly wild boar (*Sus scrofa*) and red deer (*Cervus elaphus*), live sympatrically with Iberian pigs [30] and indirect and direct risk contacts at the wildlife-livestock interface are frequent [12].

While management practices and biosecurity measures for intensive pig production are well known and protocolised [31–33], little information is available about outdoor farming systems. A detailed standardised protocol to assess and implement farm-specific preventive actions against interactions with wildlife (scalable and capable of being used in different scenarios) was recently developed for the first time in cattle [22]. Here, we adapted that protocol to extensive pig production systems and subsequently applied it to different outdoor pig farms in Spain to (1) describe the risks of wildlife-livestock interactions, and (2) outline specific biosecurity actions associated with risk mitigation. The results of this study are currently in great demand to provide objective information for disease control, particularly of ASF, at the wildlife-extensive pig production interface in Europe [8, 34].

## Results

### Characterisation of farms

The 45 selected pig farms covered a total of 25,876 ha and had a pig population of 22,577 heads. The mean farm size was 575 ha (median: 421; range 75–3009) with a mean of 11.4 (median: 10; range 1–31) plots (fenced internal divisions of farmland for grazing management) per farm, which translates into an average plot size per farm of 50.4 ha. Different types of fences were present, including traditional livestock fencing (1.2–1.5 m high; 93% of farms), which are typically made of horizontal and vertical wires 15–20 cm apart, and game fencing (2 m high but still permeable to wild boar; 7% of farms), which normally present smaller wire distances, resistant knots, and high tensile wire suitable for wildlife.

Fourteen of the 45 farms had only one production stage (one growing pig farm and 13 *montanera*-type fattening farms), while the other 31 farms integrated various stages: 27 farms included both the growing and fattening stages (five of them small commercial farms and two non-commercial family farms) and the remaining four farms also involved sows and piglets (farrow-to-finish farms; one of them was also a small farm). The average number of pigs per farm was 501 (2–1800), including all stock present in the farm during the different production stages. Beef cattle (41/45; 124 heads on average if present), sheep (22/45; mean 634 heads) and goats (7/45; mean 41 heads) were also kept on the farms surveyed. There was high heterogeneity between farms with respect to obtaining, storing, and dispensing food and water. Differences between pigs at different stages of production were more pronounced. Growing pigs requiring daily feeding and watering were reared in high densities in small plots, while during the *montanera* phase, pigs were grazed in low-density plots using exclusively natural resources. Rotational management of batches (lots) of animals or species between individual plots also varied widely, although farmers generally tended to avoid mixing species in the same plot at the same time (but they could indirectly concur on the same pastures at different times during the year) or using acorn mast for non-swine livestock. The year before the survey, anormal mortality or morbidity due to infectious diseases were recorded in 69% (31/45) of participating farms and affected pigs (19 farms), cattle (14 farms) and sheep (three farms). Notably eight farmers reported slaughterhouse condemnations in pigs because of the presence of TB-like lesions, and 20 farms did not have TB-free status in cattle. Interestingly, five out of nineteen farms where bovine had TB-free status reported slaughterhouse condemnations in pigs due to TB-like lesions.

Most of the surveyed farms (95.6%; 43/45) reported that big game was hunted in at least some areas. The average annual harvest rate was 3.8 individuals/km^2^ for wild boar (species hunted on 69% of farms) and 6.5 individuals/km^2^ for red deer (hunted on 51% of the farms). Supplementary feed was provided for wild species on almost half (48.9%; 22/45) of the surveyed farms by placing corn under large stones or in special troughs so that only wild boar could reach (60%) or spreading out in lines on the ground (40%).

### Characterisation of risk points of interaction

**Fig. 1:**
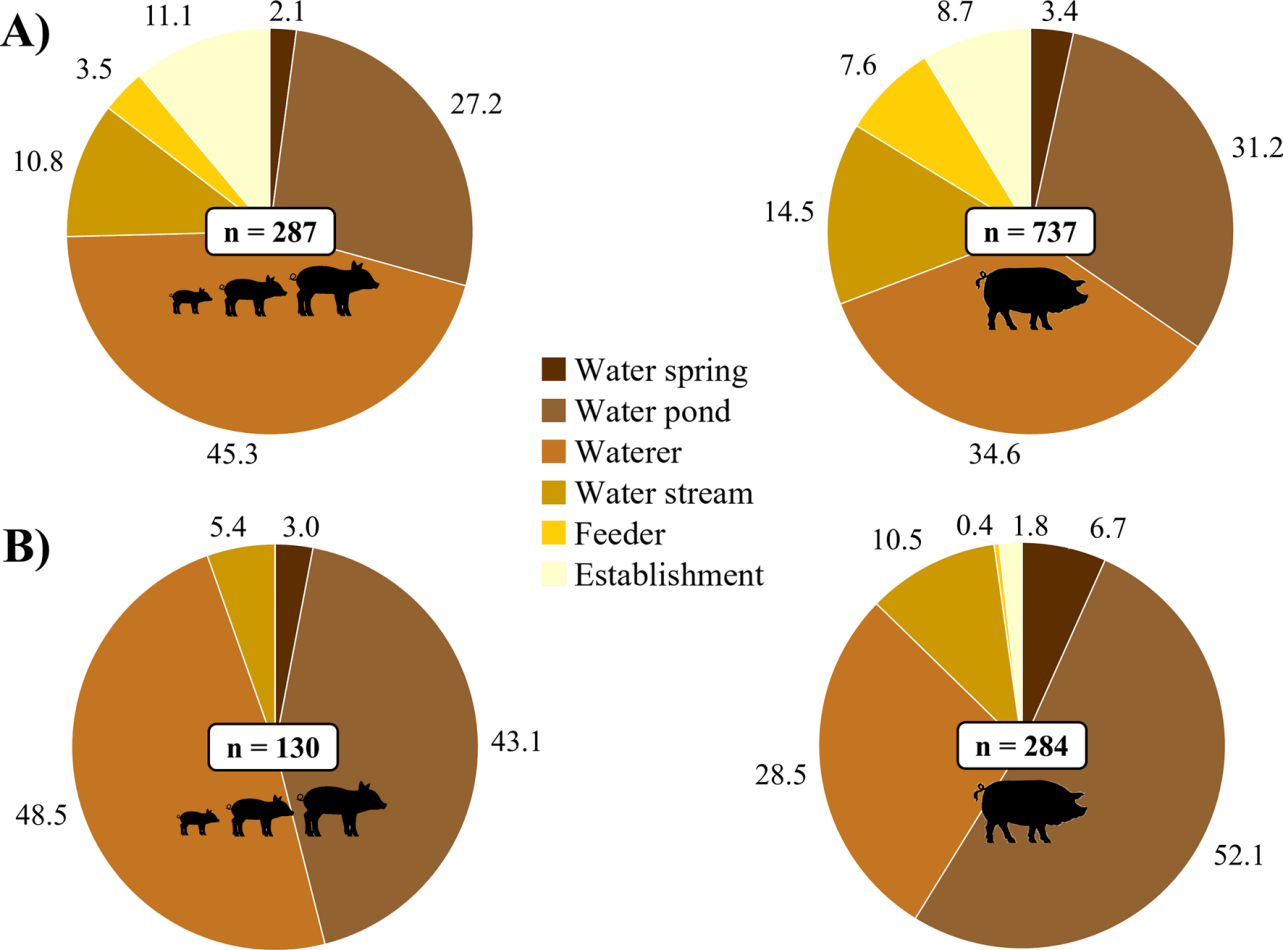
**A** Distribution of risk points by type. **B** Distribution of risk points with risk score > 3 by type. Growing (left) and *montanera*-fattening (right) stages of pig production are shown. Values represent percentages of the total number of risk points (n)

A total of 832 risk points were identified, giving an average of 18.5 (median: 17; range 4–56) per farm and 1.6 (median: 1; range 0–10) per plot. Of these, 287 (34.5%; 12.5 points/farm) were accessible to growing pigs, or to sows and piglets on traditional farms where the handling of animals was not differentiated according to stage (hereafter grouped as “growing period risk points”). Figure 1 shows the frequency of each type of point and those that were classed as high or very high risk (risk score > 3; 45.3%, see also the photographs in Additional file 1). The mean risk score was 3.4 ± 1.2 for water points (waterers or drinking troughs, ponds, streams, and springs), 2.8 ± 0.4 for feeders and fixed feeding points, and 1.2 ± 0.4 for structures associated with livestock or game management (buildings, sheds, warehouses, carcass pits, water tanks and wells, among others). More specifically, the highest mean score reported was for water ponds (4.0; n = 78), with maximum risk at 55.1% of them.

**Fig. 2:**
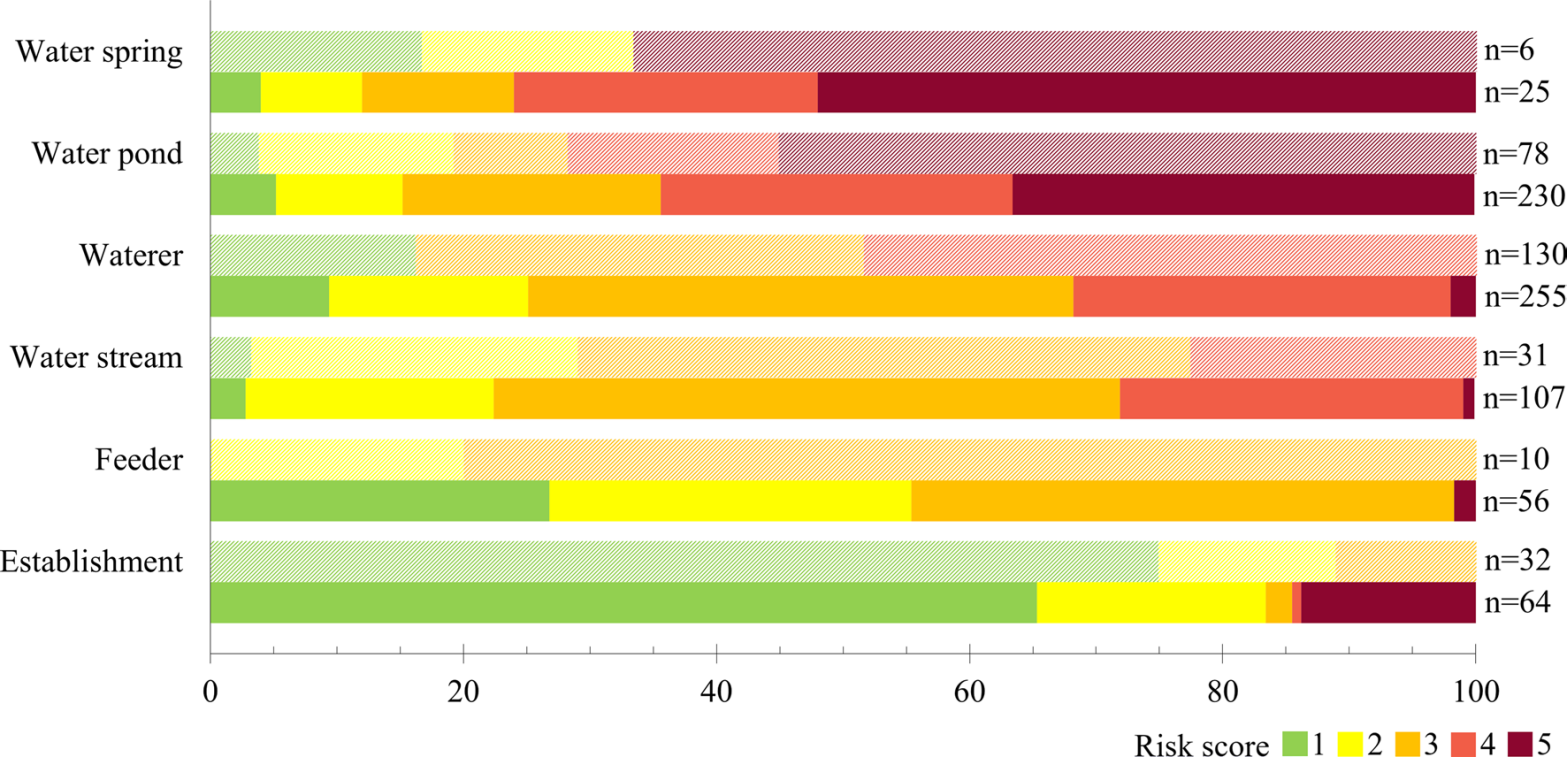
Frequency and score (1–5) of risk points grouped by type and stage of pig production. Textured light bars represent the growing period and solid dark bars the *montanera* period

In addition, 737 (88.6%; 16.8 points/farm) risk points were accessible to fattening pigs or sows on small traditional farms where all animals were handled together (hereafter grouped as “*montanera* risk points”; Fig. 1). Mean risk scores for water, feeding and management risk points were 3.3 ± 1.1, 2.2 ± 0.9, and 1.5 ± 0.8, respectively. The risk of interaction was high or very high at 38.5% of *montanera* risk points and the highest mean score was reported for water springs (4.2; n = 25) and ponds (3.8; n = 230). Finally, a total of 221 (26.6%) of the 832 risk points identified were accessible to pigs during both the growing and the *montanera* phases, so that 205 of these points (92.8%) obtained the same risk scores according to production stage. Overall, similar mean risk values (3.1 ± 1.3 and 3.1 ± 1.2) were found for points in both periods. Differences between risk score results are shown in Fig. 2.

### Management of interaction risk

Specific actions that were recommended were grouped into seven broad categories (water access, waterers, food, other livestock species, grazing, wildlife, and offal disposal) (Table 1). Considering those recommended as priority (82.7%), there were, on average, 37.1 (median: 32; range 14–113) actions to be implemented per farm, 3.2 (median: 3; range 0–29) per plot, and 2.0 (median: 1; range 0–4) per risk point. It should be noted that most of these specific priority actions (72.5%) were aimed at avoiding interspecies interactions at water points.

**Table 1:** Categorisation and description of recommended actions for interaction risk mitigation on surveyed farms

General category	Specific action	Description	Proposed as priority	Proposed as alternative
Water access control	Fence off the water point	Install livestock-proof fences to prevent livestock access to a specific water point. Bump gates can be included in this fencing, facilitating livestock access if necessary	130 (1–18)	67 (1–13)
	Remove water point	When the potential risk of interspecies interaction at the point is not offset by its utility or the efforts needed to improve it	72 (1–13)	11 (1–4)
	Fence off a water stream	Install livestock-proof fences to prevent the access of livestock to flowing water (stream, creek, river), sometimes in one plot only or on one side of the stream	53 (1–9)	17 (1–3)
	Adapt the water point	When it is not possible to install fences, usually at spring-type water points, avoid large quagmires by ensuring that the water course is channelled and can flow without stagnating with minor plumbing or masonry work	23 (1–6)	7 (1–3)
	Temporary fencing	To deter animals from crossing boundaries, install electric fences, generally made of synthetic cord with metal interwoven through it, attached to a steel fence post with a plastic insulator. Portable modular iron fencing could also be used	8 (1–3)	106 (1–18)
	Cattle-operated bump gates	Install cattle-operated bump gates with wildlife-proof fences	1	21 (1–18)
Waterer improvements	Install or modify low waterers	When more units are needed; it is recommended to provide them with features for selective-use, such as metal covers or cattle-proof tops	318 (1–33)	26 (1–9)
	Cleaning and disinfection	Routine maintenance of waterers so that clean water is available for the animals. Waterers should be dried when there is no livestock on the plot to prevent wildlife species from using them	317 (1–31)	3 (1–2)
	Install or modify high waterers	Raise waterers high enough to prevent them from being used by species other than cattle or place new units for this purpose. It is recommended to keep low waterers on the same plot dry	247 (1–31)	18 (1–6)
	Repair	Whenever waterers leak or overflow and waterlog the base. Repair should also include cementing the base (at least 1 m all around)	27 (1–3)	1
	Replace water source	When the water comes from a natural source, usually a (small) pond, replace by using the public municipal network, if available, or water obtained from a borehole	9 (1–3)	16 (1–9)
	Use disused units	Make use of waterers that are available but not in use	5 (1–3)	1
Food storage and deployment	Add or modify feeders	Install new selective feeders for cattle, or raise them to at minimum height of 1 m to prevent access by other species	9 (0–2)	
	Food storage improvement	Install or repair physical barriers to prevent livestock/wildlife access	8	
Livestock species management	Pathogen diagnosis	Check the health status of sympatric livestock and game species on the farm (depending on the epidemiological context) or those introduced from other farms of unknown health status	67	
	Spatial and temporal separation of livestock species	The aim of this action is to interrupt the natural circulation of shared pathogens between susceptible sympatric domestic species both spatially, by using different plots, and temporally, by establishing a quarantine period in the plot between the exit of one species and the entrance of another	42	
	Improve rotational grazing	Adapt grazing strategies to the environmental and epidemiological context, especially when different livestock species are present by stopping or increasing this type of handling	19	
	Remove species	When the presence of a species on the farm and the farmer’s production objectives are out of balance	15	5
	Handle or remove animals for self-consumption	Farmers do not usually handle these animals but allow them to have freedom of movement, and for feeding and drinking on the farm	9	
Grazing management	No-grazing plot (temporary)	In this case, the risk in certain areas is temporary, mainly during the summer season	54 (1–6)	17 (1–4)
	No-grazing plot (permanent)	When the risk of interaction with wildlife remains high after some action has been applied at specific points (densely wooded forest plots)	24 (1–4)	6 (1–2)
	Check and repair internal fencing	On some farms, the fences are old, broken or partially missing, resulting in poor livestock management either within or between farms, most often in the case of pigs and goats. In addition, it is common to have several openings in fences that can be used by wildlife (Fig. 4a)	23	
	Alternative grazing species	If a known pathogen is circulating and other livestock species less susceptible to that pathogen are able to graze on a specific plot (e.g., *Mycobacterium tuberculosis* complex and horses)	19 (1–5)	20 (1–4)
	Forest clearance	Remove the shrub layer to deter the presence and maintenance of wildlife and encourage livestock grazing on the most densely vegetated plots of the farm	13	2
	Reduce grazing area for piglets	Reduce the size of grazing plots for piglets during the growing period when there are potential risk points easily accessible to wildlife away from the main buildings	8	
	Install double fencing systems	Install two livestock fences a few meters apart (1–5) to prevent direct contact between animals on adjacent plots (pigs of different ages or different species)	2 (0–2)	3 (0–3)
	Avoid communal pastures	Prevent livestock grazing in communal pastures, mainly cattle	1	
Wildlife management	Improve game management	Increase hunting pressure on wild ungulates and stop practices (such as translocations) that increase population densities	45	
	Coordinate hunting plans	Agree on a coordinated hunting plan with neighbouring properties to maintain lower wild ungulate densities in order to prevent pathogen maintenance and spread to livestock	44	
	Stop use of baits	Stop artificial food supplementation for wildlife. Remove any type of wildlife feeder	23	
	Game fence	Install a 2.5 m high wildlife-proof metal fence to segregate hunting and farming activities on the farm, usually when big game is an important economic activity. It is also recommended to establish a protective barrier for specific livestock plots or risk points, and between the farm and neighbouring big game estates (perimeter fence)	13	1
Offal disposal	Improve carrion management	Increase surveillance of dead livestock in the field, change the location of animal by-product containers or improve the management of biological waste generated after hunting events	20	

Total number of individual priority or alternative actions and within-farm ranges (parenthesis) are shown

The estimated average cost of implementing the proposed priority actions per outdoor pig farm was 14,780 € (range 1061–48,615 €), giving an estimated average cost of 25.7 € per ha (range 1.3–116.4 €). More specifically, estimated average costs were 29.5 €/head (of pig) (16.0 €/head of livestock), 1294.0 €/plot and 799.4 €/risk point. Table 2 details the approximate unit costs of all materials and work needed to implement the proposed wildlife risk mitigation actions.

**Table 2:** Materials and work required to implement the proposed risk mitigation actions and approximate cost (€)/unit

Item	Unit cost
Backhoe	200 €/day
Electric fence	Power unit: 160 €
	Cable: 15 €/250 m
	Stake: 3 € (2 € fibreglass, 4 € plastic)
Feeder	275 € with assembly
Game fence	13 €/linear meter and assembly
High waterer (cattle)	200 € with assembly and base
Livestock fence	9 €/linear meter and assembly
Low waterer (pig)	150 € with assembly
Pipe and fittings	Pipe: 55 €/100 m
	Fitting: 5 €
Sack of cement (35 kg)	3.50 €
Selective bump gates	Fence: 14 €/linear meter and assembly
	Gate: 300 €
Worker	15 €/hour
10,000 L tank	3000 €

## Discussion

This research study is the first to use a systematic protocol to assess interaction risks at the wildlife-pig interface on extensive farms in Mediterranean ecosystems, and to the best of our knowledge, anywhere with respect to outdoor pig production. This standardized approach is useful for identifying relevant epidemiological features, risks and management practices on outdoor pig farms, and leads to the proposal of risk mitigation actions on a point-by-point basis.

**Fig. 3:**
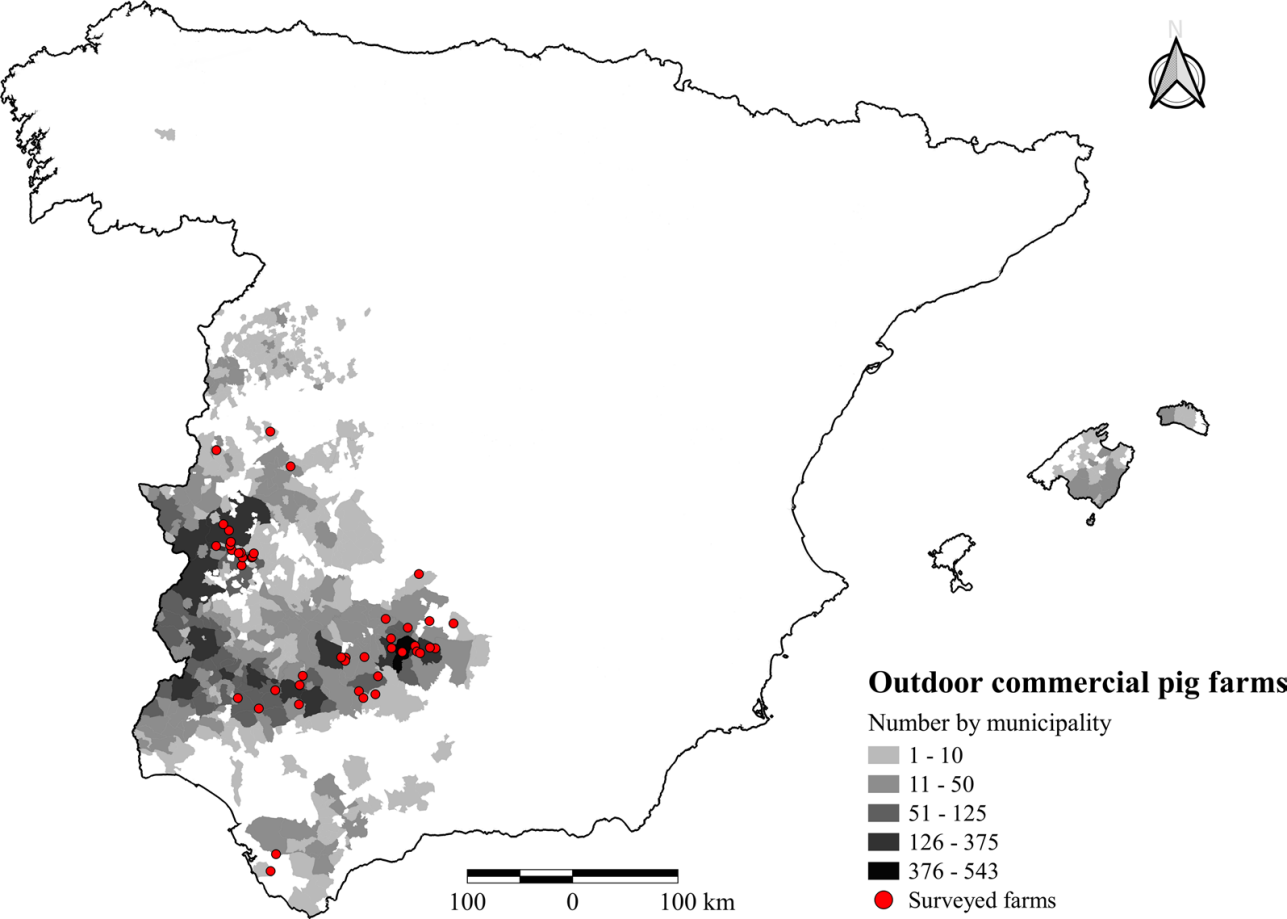
Distribution of outdoor commercial pig farms in Spain and location of the surveyed farms

The results presented here provide an overview of the wildlife-pig interface on extensive pig farms in the southwest quadrant of Spain, where most extensive pig production in Spain is concentrated. The scoring system adopted can be applied by professionals in Mediterranean environments to design specific wildlife risk mitigation actions for individual risk points or plots that can be incorporated into their general biosecurity plans. Outdoor pig farming where pigs have access to forest/woodlands and pastures/fields is currently practised in 16 (62%) and 19 (73%) of 26 European countries, respectively, according to a recent EFSA questionnaire survey [8]. Furthermore, in the current context of the spread of ASF across Europe [11, 35] and the threat posed by many other shared diseases [36, 37], adapted or extrapolated protocols that can be incorporated into integrated control and eradication strategies are in great demand. Its flexibility is demonstrated by the variety of farms among the 45 outdoor pig farms assessed in the present study (Fig. 3), ranging from non-commercial and traditional farms with pigs raised entirely outdoors to farming systems with different production stages. Different mixtures of land use and procedures for management of pigs and other livestock species were identified, as well as considerable heterogeneity of epidemiological scenarios involving wild ungulate management. Finally, the protocol is easy to adapt for professional use so that it can be implemented by technicians after a short training programme.

The higher number of risk points detected in the *montanera* stage (16.8 risk points/farm) was to be expected, given that the pigs graze over a larger area of land during this period compared to the enclosed plots used in the growing period (12.5 risk points/farm). During the *montanera* phase, pigs graze on most of the farm plots, moving between them on a cyclical basis, whereas the growing period starts within the farm facilities. During the growing period pigs are gradually given greater access to outdoor areas, although this is generally limited to a couple of plots (see sample map in Additional file 2). In many cases, the open-air plots reserved for growing pigs have previously been grazed by pigs in the *montanera* phase, which involves a risk to pigs at both production stages.

A higher average risk of interaction was expected for risk points associated with the *montanera* period, given the high frequency of (indirect) interactions observed at the wildlife-pig interface on extensive farms at this production stage [12]. Nevertheless, our results showed that the mean risk (risk score = 3.1) for pigs in the growing and *montanera* stages was similar, as most of the risk points accessible in both stages had the same score in each. In other words, whenever risk points were present, the risk score was similar in both periods. Unfortunately, there is currently a lack of information in the scientific literature about interaction risks during the growing stage in pig production. On this particular point, it should be noted that similar risk scores for the occurrence of interactions can have very different epidemiological implications depending on the stage of production. For instance, effective pathogen transmission between species at a high-risk water point can be more likely during the growing period than the *montanera,* because in the former, there is less water available, animal density is higher and therefore daily water use by pigs increases. Furthermore, an infection acquired during the growing period can spread within or between farms (and to other susceptible sympatric species) if animals are mixed between batches or are moved to other farms for fattening. Decision-making about the design and prioritisation of risk mitigation actions should therefore take an integrative view and consider not only the assigned risk score, but also the information gathered through the on-farm questionnaires.

The relative frequency of risk points by type was also very similar between pig production stages (Fig. 1): > 80% of interaction risk points were water points, while feeding and management accounted for < 10% in both cases. These proportions are also in line with those previously observed on extensive cattle farms in the study area [22]. Water points have been widely reported as a risk factor for the occurrence of interactions at the wildlife-livestock interface in Mediterranean ecosystems [9, 11, 12, 38, 39]. They are recognised hotspots for the maintenance and transmission of multi-host pathogens in outdoor farming systems [40, 41]. The epidemiological relevance of water points in the epidemiology of transmissible diseases has been shown to increase when water resources are limited, mainly in the summer season [39, 42–44]. However, even though the availability of water may not be a limiting factor during the *montanera* period (October-February), which partly coincides with the rainy season in Spain, it should not be ruled out as a potential risk factor, particularly during dry years. Around 75% of the pigs' daily water intake is closely associated with eating bouts and feed consumption [45]. During the *montanera* phase, pigs spend around 6–7 h a day grazing and so drink water continuously [46]. In dry years, both the number and size of available water points decrease, and the water intake of pigs is concentrated around those that are still there. Most of the specific actions (74.6%) therefore were focused on controlling access to water points and on improving their fundamental features (Table 1). Risk mitigation was addressed mainly by (1) using fencing to separate species using water points [18] and (2) replacing water sources with more hygienic options such as waterers of different heights and/or with protective elements to try to make them species-specific. The need to implement the proposed actions was particularly emphasised at water springs and ponds, where the risk of interaction between domestic and wild species was seen to be greatest (Fig. 2). Specific behaviour and farm resource use by different wild ungulates (e.g., Carrasco-García et al. [42]), together with their specific ability to cross barriers (e.g., Barasona et al. [18]), determined that the proposed actions depended on the specific wildlife and livestock species involved.

**Fig. 4:**
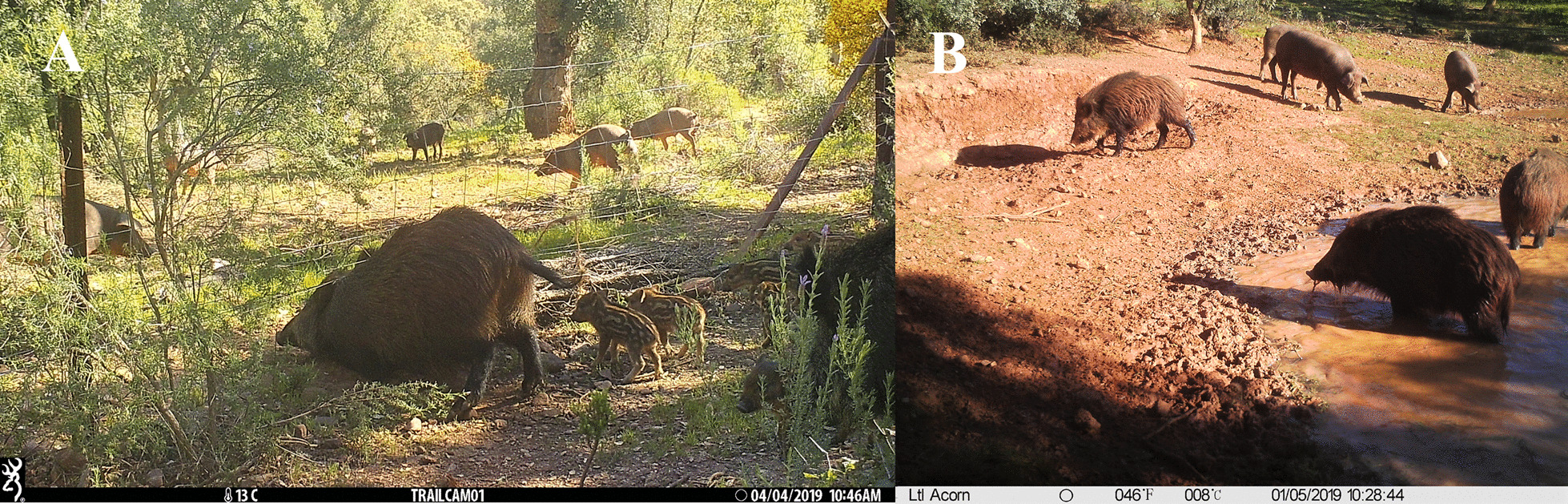
Camera trap pictures of interactions between Iberian pig and wild boar. **A** The ability of wildlife to cross livestock-proof single fences in a *Quercus* spp. natural feeding area, and **B** the potential epidemiological implications of the shared use of water points are indicated

In terms of interspecies interactions, feeding points and facilities associated with pigs (growing phase), livestock and game management were less significant in both number and assigned risk scores (Figs. 3 and 4). This is consistent with the low rate of wild ungulate detection at outdoor feeders and facilities on different extensive farms in Mediterranean environments in Spain [42]. Feeding points for growing pigs were placed inside the pig buildings (a major difference compared to water points, which were normally outside) and were not accessible to other domestic or wild species (risk score = 0). In general, outdoor feeding paddocks in the growing stage were also enclosed by a solid wall made of stones, cement blocks or brick, leading to the minimisation of the risk of interspecies interaction. With respect to the *montanera*, most of the common feeders (often empty) were used by farmers to feed other livestock or game species rather than to fatten pigs and in other season, since pigs mostly fed on natural resources such as acorns and grass while grazing in the *dehesa during this production stage*. Indeed, during the *montanera*, other livestock were normally kept in separate plots or inside fenced pens, so that the mast was exclusively used by fattening pigs. At other wildlife-livestock interfaces, selective feeders were recommended as a risk mitigation priority at feeders with significantly high scores [47]. In the present study, we acknowledge that the scoring system may underestimate the risk of natural resource feeding, although this aspect was partially addressed in the section on livestock and game management. Grazing plots for pigs during the *montanera* are savannah-like habitats, made up mainly of *Quercus* spp. oak trees and pastureland (typical of the *dehesa*). These plots have previously been described as attractive to wild ungulates [42], with the risk of interactions with livestock being distributed over a wider area than at specific feeding points. Kukielka et al. [9] monitored food placed on the ground for game baiting and grazing pastures in acorn fields in a similar setting, and the rate of detection of wild ungulates was even higher than at water sites (revealing that epidemiological risks are still present). Other studies carried out to characterize this wildlife-livestock interface at a finer scale also showed the importance of shared feeding resources for the occurrence of interspecific contacts, mainly indirect [12, 43].

The risks associated with livestock and game management were addressed by combining the on-site evaluation and open questions in the questionnaire (Additional file 3). Specific actions related to certain farming and hunting practices were classified into four general categories (Table 1). These measures were mostly recommended as priorities because many of them, like removing livestock species or reinforcing perimeter fencing, directly eliminated or greatly reduced the risk score at many other points. Wildlife risk mitigation and biosecurity actions that are common to different livestock species (such as cattle and pigs) have been previously discussed, monitored, and evaluated to assess their practical feasibility, acceptability and to promote their proactive acceptance by farmers [22]. It should be noted that the implemented actions need to be effective and compatible over different stages of the pig production cycle, and also take other uses of farming land into account. Big game was the most challenging issue to be tackled in this respect, mainly because it represented a profitable source of revenue on most farms (95.6%) and generated significant economic incomes on many of them. At the same time, wild ungulate populations in general, and particularly wild boar, represent a relevant threat to sympatric outdoor pigs for transmission of diseases such as TB in Mediterranean Europe [7] or ASF in central and eastern regions of Europe [8]. Consequently, it has always been recommended to improve game management and coordinate hunting plans to control wild ungulate populations and in fact it has always been suggested that risky management practices such as wildlife baiting should be discontinued. Moreover, when it was shown that trade-offs between outdoor farming and big game were incompatible because of the potential number of risks envisaged, we highlighted the need to choose one of the activities exclusively and to shift the risk mitigation actions to the other one. Finally, management of livestock or game offal was found to be deficient on almost half (44.4%) the farms. Although up-to-date regional [48–50] and national [51, 52] regulations do currently exist and have been shown to be effective for risk reduction [53], the importance of including farmers in the study area in specific awareness campaigns on this matter is highlighted.

From an economic perspective, the estimated average cost of implementing the proposed priority actions in outdoor pig farming was slightly higher per farm, plot, and risk point, but lower per hectare and head of livestock than those recommended for extensive cattle in the same study area [22]. This reflects the greater number of species and animals present on the surveyed farms and the need to improve species segregation. The increase in estimated cost may be due to the more general recommendation of fencing tools, which are more expensive, as preferred options to mitigate wildlife risks and segregate animal species at specific points (Table 1). Although this approach is expensive, it is in line with the current legislation for TB control in these complex scenarios in Spain [51] and also with the scientific opinion on ASF and outdoor pig farming in affected areas in Europe [8]. Nonetheless, even though fences can limit wild boar movements, they are not 100% effective, mainly due to the presence of streams and other points of vulnerability (Laguna et al., under review). Further monitoring of the proposed biosecurity measures and acceptance by farmers are necessary for the standardisation of this on-farm risk-based assessment of outdoor pig farming.

## Conclusions

This research describes, for the first time, a systematic approach to on-farm risk assessment at the wildlife-pig interface. It is able to define the epidemiological characteristics of outdoor pig farms, their management, and associated risks using a standardised proposal of risk mitigation actions to avoid or prevent specific risks associated with interaction points. The flexibility of this protocol makes it easy to transfer to professionals and to adapt to extensive (outdoor) production or epidemiological systems in other European regions. The subsequent monitoring of the proposed biosecurity measures and, above all, increasing their acceptance by farmers are necessary steps for the further development of ASF (and other transboundary diseases) control programmes. This approach for extensively reared pigs represents a key practical step towards the preventive and integrative health management of disease at the interface with wild ungulates. However, such an integrative approach requires the commitment of all involved stakeholders, including wildlife decision-makers, managers, and hunters, to control the transmission of shared diseases.

## Materials and methods

### Study area

The study was carried out in southwestern Spain, a large area characterized by the *dehesa* agroforestry system interspersed with Mediterranean forest, where different land uses such as farming, agriculture and/or recreational activities (including hunting) are simultaneously exploited [29]. Physical barriers do not necessarily exist or may be placed exclusively to limit livestock movements, which means that wild ungulates can easily cross them [42]. This increases the opportunities for direct (that is, very close physical contact between wild ungulates and pigs) or, more often, indirect interactions (by sharing resources like water or food within predefined time windows) between livestock and wild ungulates (Fig. 4; see also [12]). The climate in the study area is characterized as Mediterranean, in which the dry season (June–September) is critical for the availability of natural resources therefore increasing the risk of interactions between wildlife and livestock. Another critical period for interactions is autumn when *Quercus* spp trees produce acorns (see below).

### Extensive pig production in the Iberian Peninsula and farm selection

Iberian pigs (and crossbreeds) are typically linked to the southwestern quadrant of Spain (and adjacent areas of Portugal). The production of these animals constitutes a professional and commercial sector (not a backyard system) in which they are mainly raised under extensive conditions. In recent decades, the traditional extensive pig sector has undergone a profound transformation, leading to a great diversity of farming systems [54]. The tendency on more technified professional farms is to differentiate between production stages (breeding-gestation, lactation-weaning, growing, and fattening). Enclosed housing, with little or no access to the outside, is used for sows and piglets until weaning (up to 23 kg). During the growing period (up to 110 kg), the piglets are usually reared on *dehesa* pasture connected to open buildings or feeding paddocks. The final fattening phase (up to 160–185 kg), known as the *montanera*, starts in autumn when the pigs remain outdoors in the *dehesa* to feed on acorns and grass. Nevertheless, traditional farrow-to-finish farms, small commercial farms (< 25 fatteners and < 5 sows) and non-commercial family farms (< 5 fatteners for self-consumption) continue to be prominent despite the transformation in the extensive sector [55].

In this study, we focused on Iberian pig farms where pigs have access to the outdoor area in the *dehesa* (outdoor pig farms). A total of 45 farms (Fig. 3) were selected by convenience sampling according to the farmers’ interest. The aim was to include a wide variety of outdoor systems, epidemiological contexts, and handling procedures. All farms were audited between 2015 and 2017.

### On-farm study design

To carry out this study, we adapted the protocol developed by Martínez-Guijosa et al. [22] for cattle farming to the particular features of the extensive pig production system in the Iberian Peninsula. The main characteristics distinguishing the cattle and pig production systems are the different production stages, differential use of the farmland, and the shorter life cycle of the pigs (see above), most of which are slaughtered and replaced annually. Replacement implies regular movement of animals from one farm to another and/or between production stages within the farm.

General information was first obtained from farmers through telephone interviews and/or the Veterinary and Forestry Authorities so that a preliminary characterisation of each farm could be made before the visit. Data on location and size, types of fencing, land uses, domestic and wild species management, hunting bags, animal movements and health records were collected, as well as data about neighbouring properties whenever possible. The information was georeferenced and appropriately scaled farm perimeters were drawn on maps (1:10,000) for use in subsequent audits.

More comprehensive information about each selected farm was gathered through on-site audits. A structured questionnaire was filled in with the farmers to complete the characterisation of the farms and to identify potential sources of interspecies interactions that are considered risk points and risky management practices for both domestic and wild species. In parallel, all relevant features posing a risk for the transmission of shared infections were recorded on the printed map. Both the questionnaire and the map (example), as well as other useful fieldwork documents and instructions for completion can be found in Additional files 2, 3, 4.

Once the audits were completed, all potential risk points for interaction that had been identified were visited and assessed (with a score from 0 to 5), following the criteria described in Martínez-Guijosa et al. [22]. A multidisciplinary team consisting of three experienced observers carried out all the visits to avoid possible biases in risk scoring. A score of 0 was reserved for points that pigs never had access to (although they could be used by wildlife), while scores 1–5 were assigned progressively to pig facilities, taking into account opportunities for access, the presence or signs of wildlife or other livestock species, as well as the specific characteristics of the points/practices themselves. Points that scored 0 were removed from the analysis, so that risks (in a broad sense) were defined as those points and actions where interactions between pigs and other domestic and wild ungulates could occur.

All the information gathered through questionnaires and audits were written on paper forms at farms. Then, the collected information was converted into electronic format as spread sheet (Excel), which were the basis for subsequent statistical descriptive analysis. The highest resolution level of data was at risky point level within farm, indicating their characteristics and proposed actions. Descriptive statistics for central tendency and dispersion were obtained at both farm and risk point levels. Finally, based on the previous experience of the research team [12, 18, 22], farmers were informed of specific measures to mitigate the potential risk of interaction and pathogen transmission on each study farm and risk point. A set of general and specific actions were designed and prioritized by risk based on the estimated risk score and the ability of each measure to prevent interactions at the wildlife-domestic interface. The cost of implementing the proposed priority actions was estimated for each farm, using the approximate unit costs of all materials and work required for implementation.

## Supplementary Information

**Additional file 1**. Photographic appendix showing examples of high and very high-risk points

**Additional file 2**. Sample map

**Additional file 3**. Questionnaire

**Additional file 4**. Basic instructions and fieldwork documents

## Data Availability

The datasets used and/or analysed during the current study are available from the corresponding author on reasonable request.
