# Supplementary material for: Characterization and management of interaction risks between livestock and wild ungulates on outdoor pig farms in Spain
Source: Porcine Health Manag. 2022 Jan 5;8:2. doi: 10.1186/s40813-021-00246-7 (PMC8734068; doi:10.1186/s40813-021-00246-7)
Supplement: Supplementary file 1 — Additional file 1. Photographic appendix showing examples of high and very high-risk points [file 40813_2021_246_MOESM1_ESM.docx]

**Additional file I.** Photographic appendix showing examples of high and very high-risk points.

**Water ponds**

| **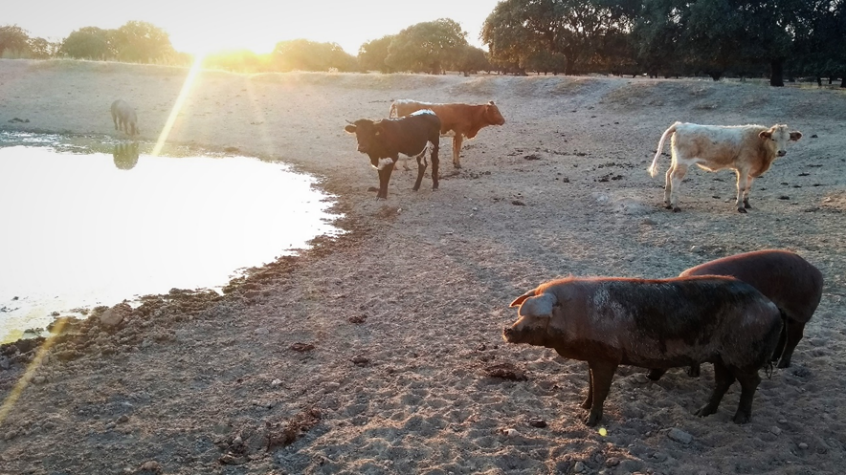**  **Risk score = 5** | Permanent pond of 19 x 18 m. Unfenced. Shared use by pigs and cattle. Signs of red deer in the surroundings |
| --- | --- |
| **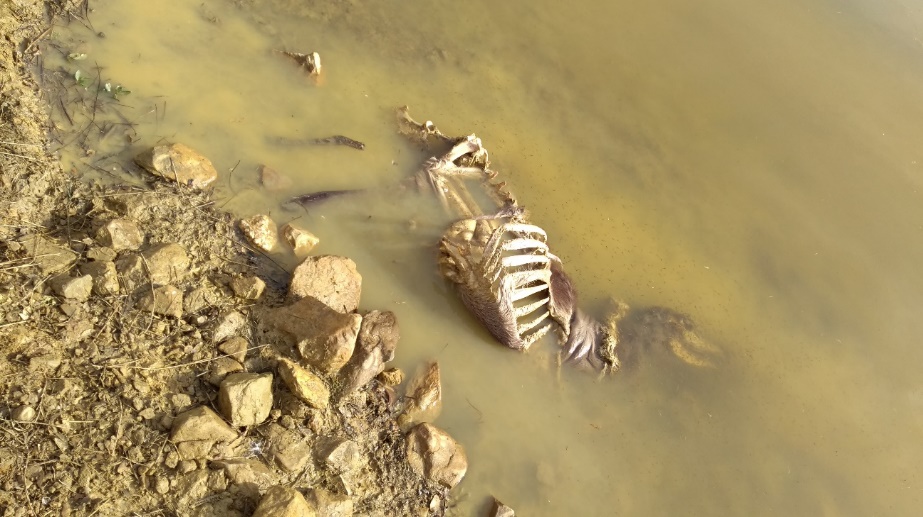**  **Risk score = 5** | Red deer carrion into a water pond accessible to fattening pigs |
| 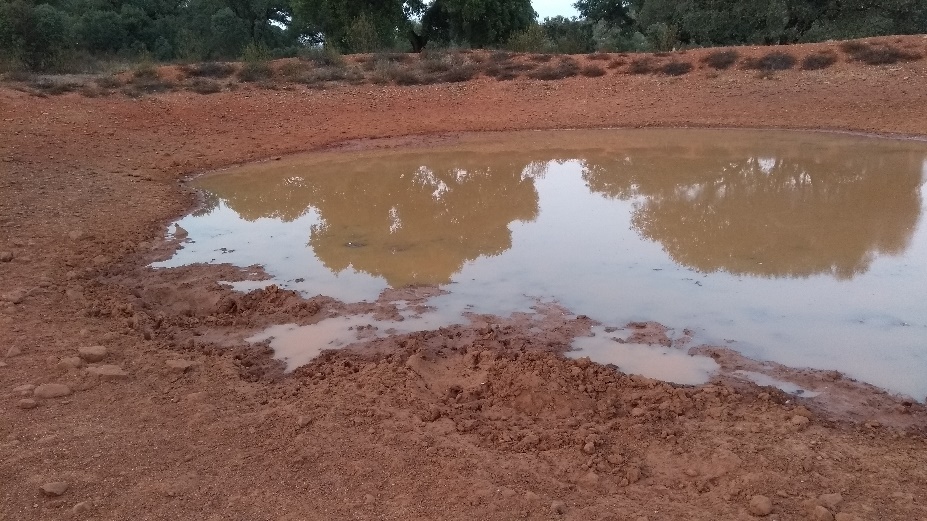  **Risk score = 5** | Permanent pond of 17 x 23 m. Unfenced. Accessible to both growing and fattening pigs. All signs corresponded to wild ungulates |

**Water springs**

| **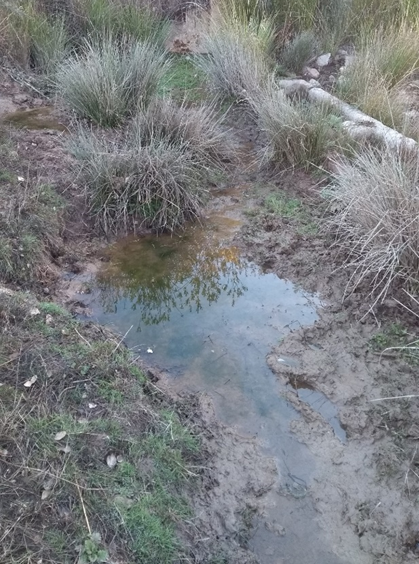**  **Risk score = 5** | Permanent flooded spring of 1.5 x 4 m that extend a muddy area to one side. Unfenced. Used by pigs while fattening, sheep during spring and summer and game species throughout the year. Abundant signs of wild boar and red deer |
| --- | --- |

**Water streams**

| **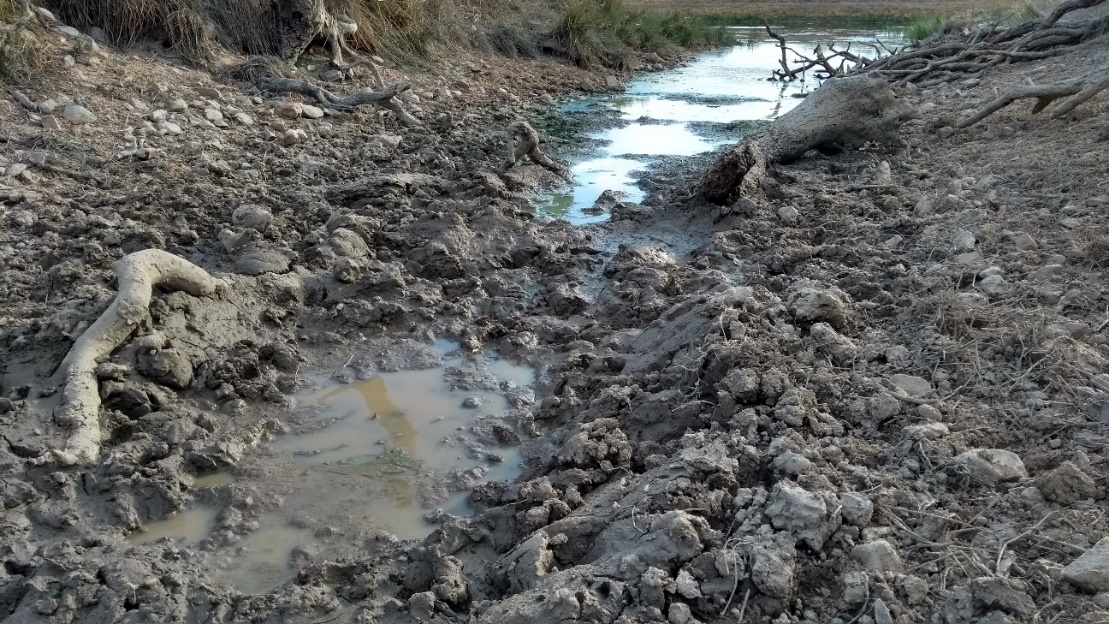**  **Risk score = 5** |
| --- |
| Seasonal muddy pools and backwaters formed in summer on a large stream that maintains water all year round. Unfenced, it affects to different plots of the farm. All signs corresponded to wild ungulates |

**Waterers**

| 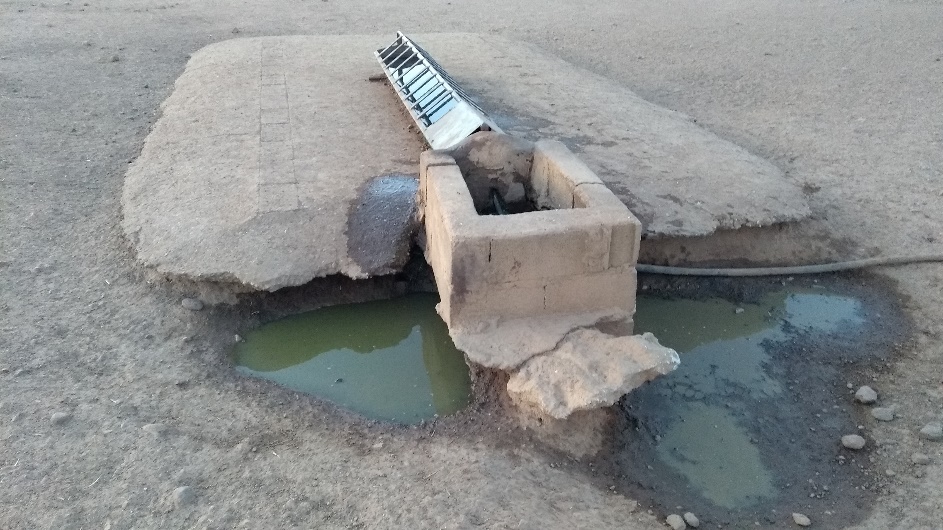  **Risk score = 4** | Ground-level watering trough with flooded area around. Unfenced. Used by growing pigs |
| --- | --- |
| **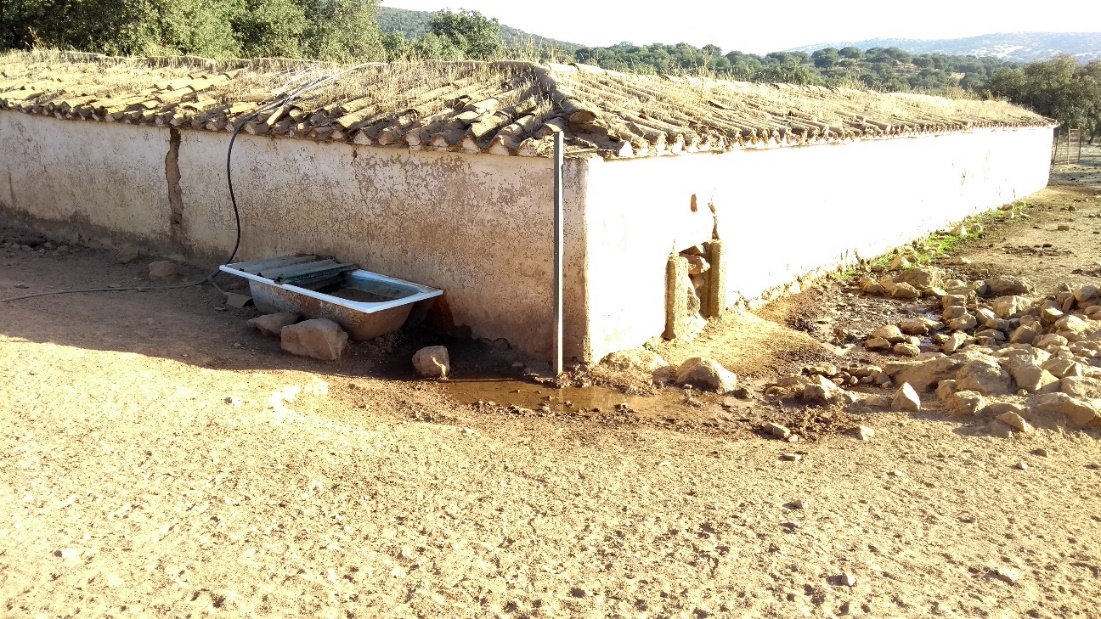**  **Risk score = 4** | Mid-high-level watering trough with flooded area around. Unfenced. Used by cattle and fattening pigs. Signs of wild ungulates |
| **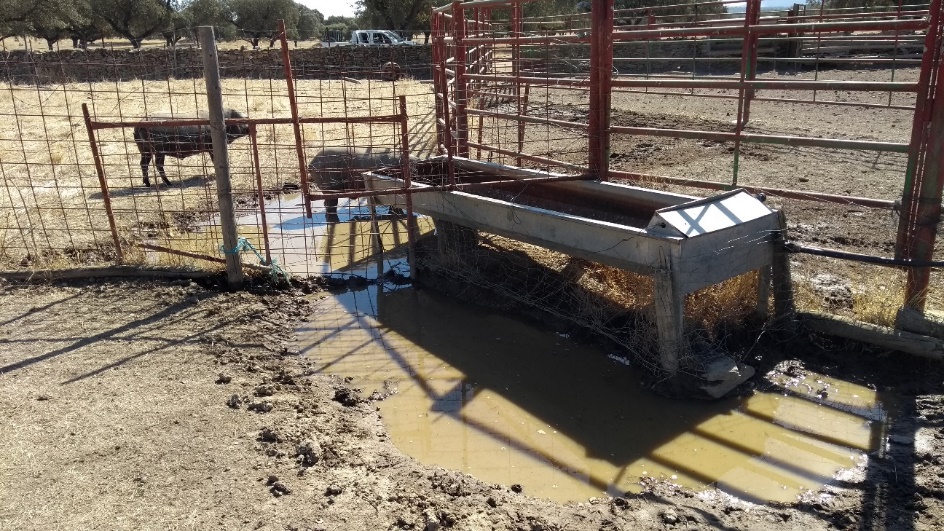**  **Risk score = 4** | High-level watering trough selective for cattle with flooded and muddy area around. Fenced, but accessible to pigs and wild species |

**Feeding points**

| **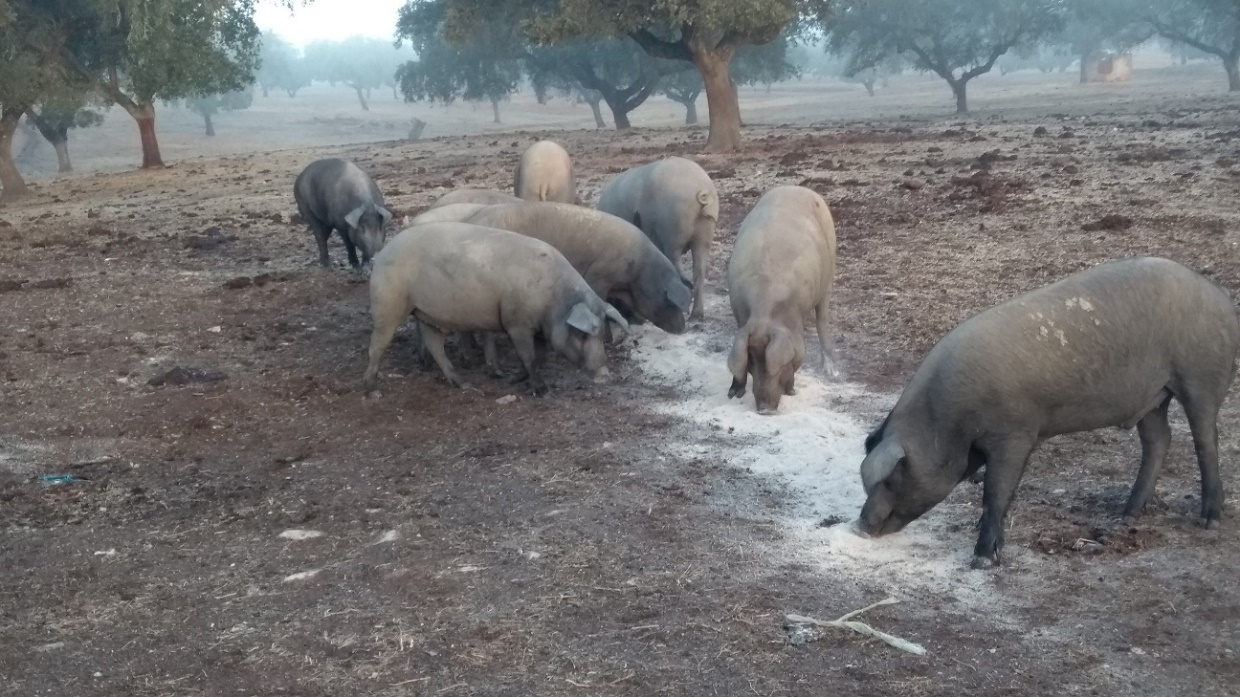**  **Risk score = 4** | Random feeding point on the ground for non-commercial self-consumption outdoor pigs. Plot used permanently by cattle. Presence of wild ungulate signs in the plot |
| --- | --- |
| **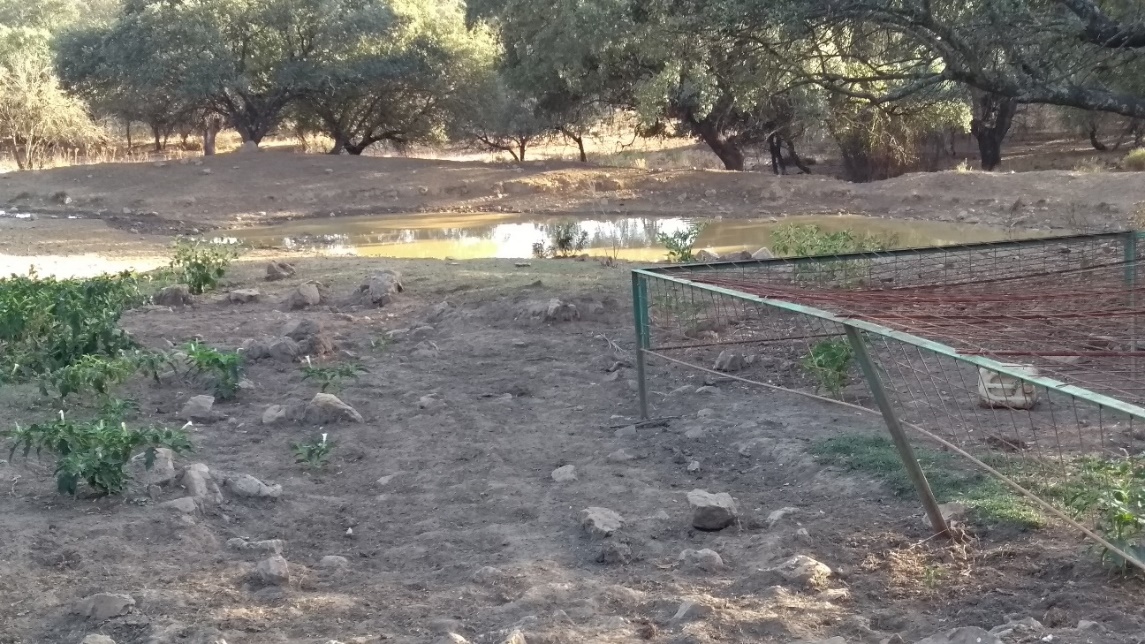**  **Risk score = 5** | Feeding trough designed to a selective use for piglets. Here, it was used for wild boar baiting within a *montanera* plot and placed just join to the exclusive available watering site. Abundant signs of wild boar and red deer throughout the feeding and watering area |

**Establishments**

| **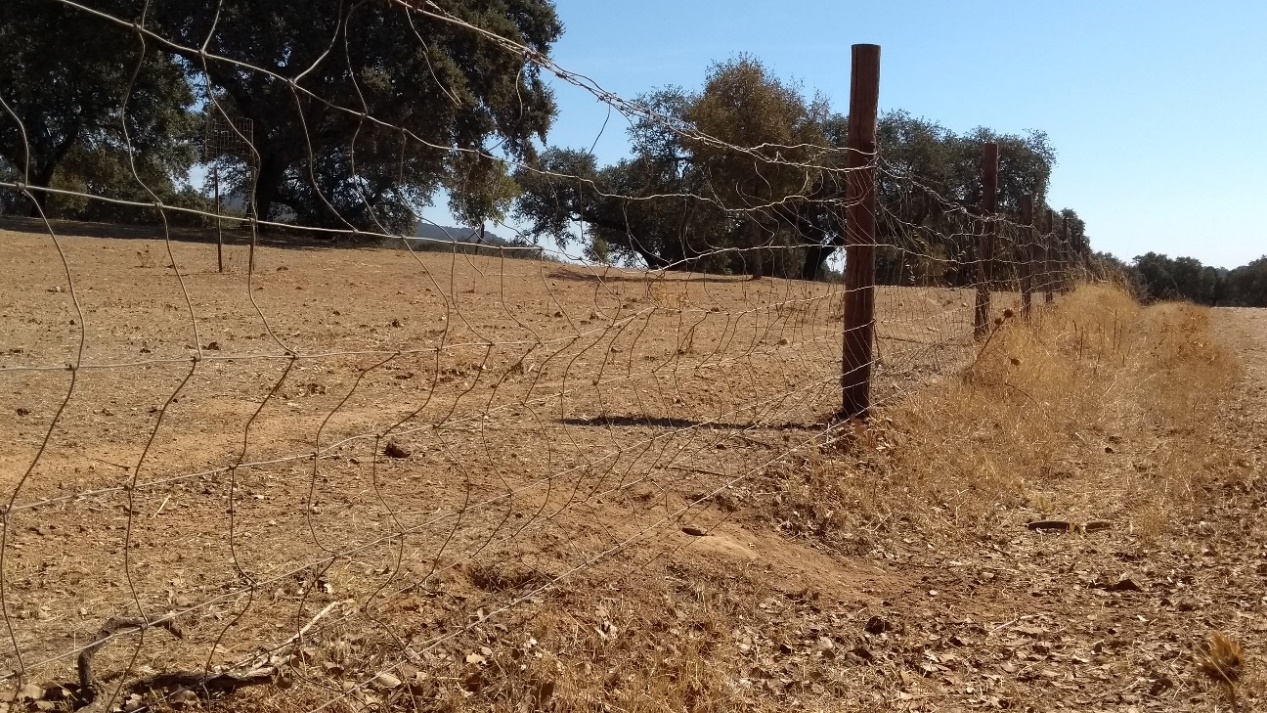**  **Risk score = 3** | |  |
| --- | --- | --- |
| Perimetral fencing between two mixed farming and game properties. It is noteworthy the bad status of maintenance. Signs under and over the fence are indicatives of wild boar and deer crossing, respectively | |  |
| **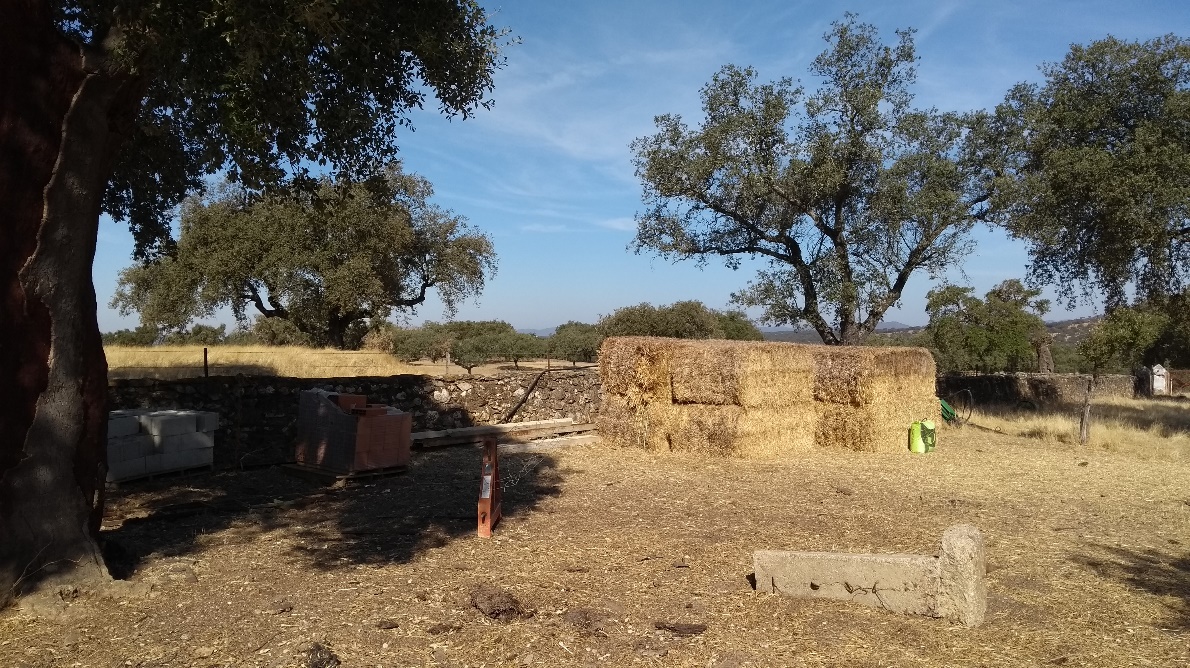**  **Risk score = 4** | Storing of hay without environmental protection in a plot fenced by single solid (masonry) fence. Easily accessible to domestic animals grazing at this plot and wild ungulate species, mainly red deer | |
