# Supplementary figures and images for: Characterization and management of interaction risks between livestock and wild ungulates on outdoor pig farms in Spain
Source: Porcine Health Manag. 2022 Jan 5;8:2. doi: 10.1186/s40813-021-00246-7 (PMC8734068; doi:10.1186/s40813-021-00246-7)

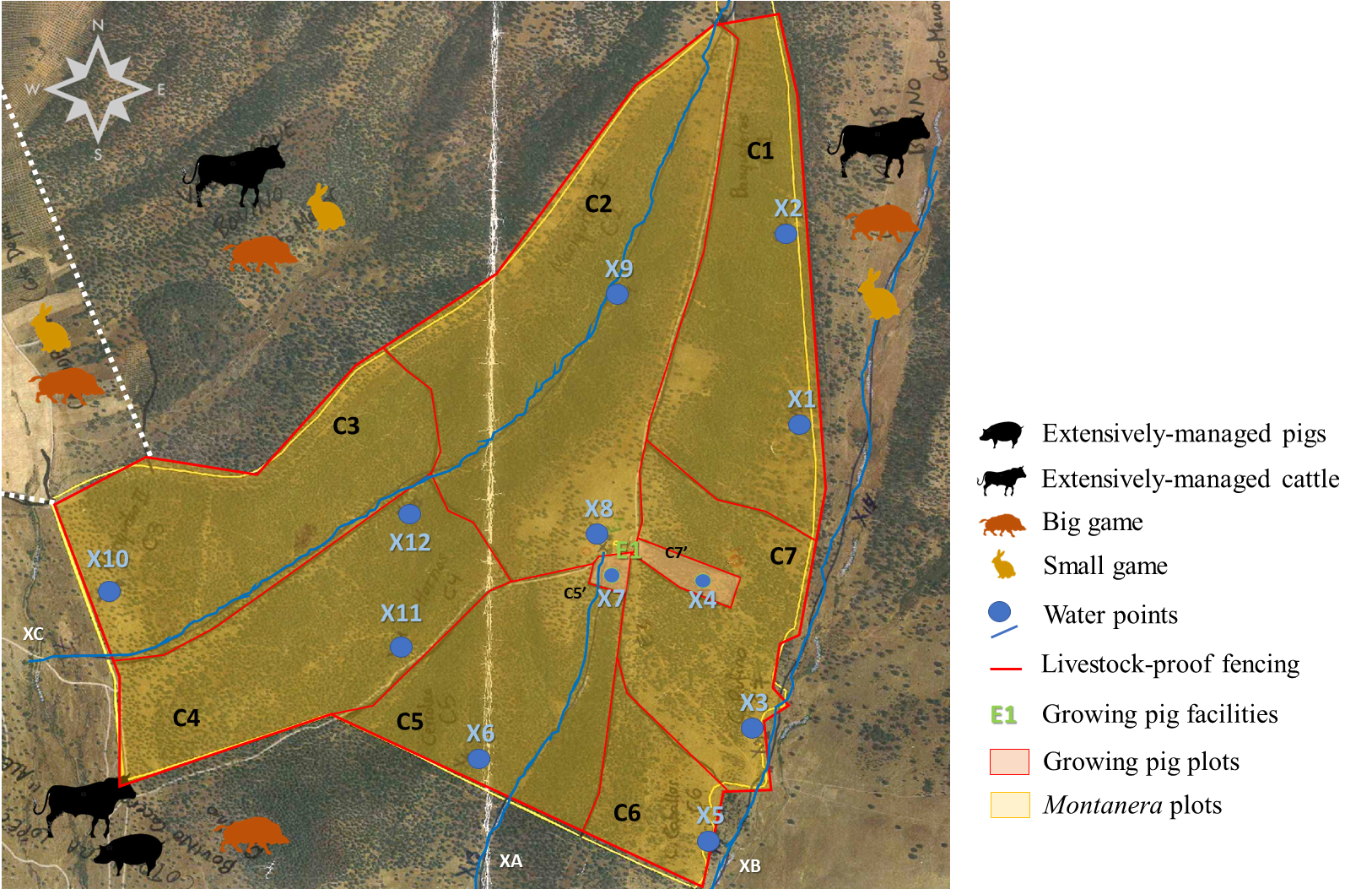

Supplement: Supplementary file 2 — Additional file 2. Sample map [file 40813_2021_246_MOESM2_ESM.tif]
