# Supplementary material for: Characterization and management of interaction risks between livestock and wild ungulates on outdoor pig farms in Spain
Source: Porcine Health Manag. 2022 Jan 5;8:2. doi: 10.1186/s40813-021-00246-7 (PMC8734068; doi:10.1186/s40813-021-00246-7)
Supplement: Supplementary file 3 — Additional file 3. Questionnaire [file 40813_2021_246_MOESM3_ESM.docx]

**Additional file III.** Questionnaire

1. **Personal information**

Farm name:

REGA code (General Registration of Livestock Farms):

Municipality:

OCA (District Agricultural Office):

Name of the farmer/contact: Position:

Phone number: E-mail:

Address:

Worker responsible for farm animals: Worker’s phone number:

Farm’s veterinary practitioner: Vet’s phone number:

ADSG (Livestock Health Defence Group):

Number and position of workers:

1. **Farm characteristics and land management**

Total surface (ha):

Farm surface (ha):

Pastureland surface (ha):

Bushland surface (ha):

Limiting perimeter (m) with game estate:

Livestock: type of production, number of heads and breeds

Porcine: reproduction / growing / fattening (underline what is present)

Bovine: Ovine: Caprine:

Other domestic species:

Livestock units and batches/herds (number, point on the map):

Watering and feeding (include references on map and plot sheet)

Number of waterers:

Number of water ponds:

Number of water springs:

Number of water streams:

Number of feeding points:

Food type:

Food origin:

Food storage:

Description (write feeding strategy and plot management).

Offal disposal

Livestock carrion (number of animals and species). Identify each species with a number or letter to identify them in the next step (offal disposal type and %).

| Species / Month | 1 | 2 | 3 | 4 | 5 | 6 | 7 | 8 | 9 | 10 | 11 | 12 |
| --- | --- | --- | --- | --- | --- | --- | --- | --- | --- | --- | --- | --- |
|  |  |  |  |  |  |  |  |  |  |  |  |  |
|  |  |  |  |  |  |  |  |  |  |  |  |  |
|  |  |  |  |  |  |  |  |  |  |  |  |  |
|  |  |  |  |  |  |  |  |  |  |  |  |  |
|  |  |  |  |  |  |  |  |  |  |  |  |  |

Official removal (__%) Burial (__ %) Scavengers (__%) Vulture feeding point ( __%)

Official removal (__%) Burial (__ %) Scavengers (__%) Vulture feeding point ( __%)

Official removal (__%) Burial (__ %) Scavengers (__%) Vulture feeding point ( __%)

Official removal (__%) Burial (__ %) Scavengers (__%) Vulture feeding point ( __%)

Official removal (__%) Burial (__ %) Scavengers (__%) Vulture feeding point ( __%)

Wildlife carrion management (What happens to game waste?):

Sanitary problems recorded the last year (indicate type and species):

1. **Wildlife**

Is the farm a (big or small) game estate? Estate number:

Does the farm limit with big game estates? How many?

Number (or names) of the adjoining game estates (map):

Number of hunted deer/season: Number of hunted wild boar/season:

Other big or small game (number/season):

Usual wildlife species, add others:

| Species | Presence | | | | | | |
| --- | --- | --- | --- | --- | --- | --- | --- |
|  | Daily | Weekly | Monthly | Very sporadic | Never | Location* |  |
| Red deer |  |  |  |  |  |  |  |
| Wild boar |  |  |  |  |  |  |  |
| Roe deer |  |  |  |  |  |  |  |
| Mouflon |  |  |  |  |  |  |  |
| Fallow deer |  |  |  |  |  |  |  |
| Badger |  |  |  |  |  |  |  |
|  |  |  |  |  |  |  |  |
|  |  |  |  |  |  |  |  |

*Point on the Map

Baiting for wild ungulates on the farm. Describe wildlife baiting strategy (use Map).

Sanitary problems recorded the last year (indicate type and species):
