# Supplementary material for: Characterization and management of interaction risks between livestock and wild ungulates on outdoor pig farms in Spain
Source: Porcine Health Manag. 2022 Jan 5;8:2. doi: 10.1186/s40813-021-00246-7 (PMC8734068; doi:10.1186/s40813-021-00246-7)
Supplement: Supplementary file 4 — Additional file 4. Basic instructions and fieldwork documents [file 40813_2021_246_MOESM4_ESM.docx]

**Additional file IV.** Basic instructions and fieldwork documents.

1. **Basic instructions**

ON-FARM MAP

- Check perimeter.
- Indicate name and uses of limiting farms.
- Delimiting indoor fencing, indicating uses.

PLOT SHEET

- - Type of fencing: game (g) and livestock (l).
  - Indicate buildings, warehouses, and different types of facilities on the farm.
  - Feeding points (label them with a number that corresponds to the table).
  - Indicate water sources (label with numbers that corresponds to the table).
    - Permanent and seasonal water ponds and springs.
    - Permanent and seasonal streams/rivers, indicating areas of water permanence in dry season.
    - Waterers or water troughs.
    - Water reservoirs.
    - Other (indicate).

FIELD WORK

- Check fencing status of perimeters and enclosures, especially in a priori areas of risk.
- Check every water and feeding point (use “risk sheets”).
- Indicate observations, diagnosis of use and measures to be proposed in the table.
- Check every livestock or game facility.
- Take pictures of every risk point.
- Use photo-trapping to evidence interaction risks.

1. **Plot sheet**

| Plot | Type of fencing | Fencing status and height | Land uses (pay attention to periphery or bushland limits)* |
| --- | --- | --- | --- |
|  |  |  |  |
|  |  |  |  |
|  |  |  |  |
|  |  |  |  |
|  |  |  |  |
|  |  |  |  |
|  |  |  |  |

* Livestock/Game/Agricultural. Describe: pastures, facilities, bushland, crops (type). Use also the Map.

1. **Risk point sheet**

| Type | Map reference | Size (m) | UTM | Sample/camera |
| --- | --- | --- | --- | --- |
|  |  |  |  |  |
|  | Description/diagnostic: | | | |
|  |  |  |  |  |
|  | Description/diagnostic: | | | |
|  |  |  |  |  |
|  | Description/diagnostic: | | | |
|  |  |  |  |  |
|  | Description/diagnostic: | | | |
|  |  |  |  |  |
|  | Description/diagnostic: | | | |
|  |  |  |  |  |
|  | Description/diagnostic: | | | |
|  |  |  |  |  |
|  | Description/diagnostic: | | | |
|  |  |  |  |  |
|  | Description/diagnostic: | | | |
